# Role of Immune Cells and Immunotherapy in Multiple Myeloma

**DOI:** 10.3390/life14040461

**Published:** 2024-04-01

**Authors:** Vijay Radhakrishnan, Upendarrao Golla, Avinash Kundadka Kudva

**Affiliations:** 1 Department of Surgery, Ellis Fischel Cancer Center, Roy Blunt NextGen Precision Health Institute, University of Missouri, Columbia, MO 65212, USA; vrfgz@missouri.edu; 2 Department of Medicine, Division of Hematology and Oncology, Pennsylvania State University College of Medicine, Hershey, PA 17033, USA; ugolla@pennstatehealth.psu.edu; 3 Department of Biochemistry, Mangalore University, Mangalagangothri, Mangaluru 574199, India; 4 Department of Pediatrics, Pennsylvania State University College of Medicine, Hershey, PA 17033, USA

**Keywords:** multiple myeloma, immune cells, bone marrow microenvironment, proteasome, CAR T-cell, immune escape, immune checkpoint inhibitors, immunotherapy

## Abstract

The clinical signs of multiple myeloma, a plasma cell (PC) dyscrasia, include bone loss, renal damage, and paraproteinemia. It can be defined as the uncontrolled growth of malignant PCs within the bone marrow. The distinctive bone marrow milieu that regulates the progression of myeloma disease involves interactions between plasma and stromal cells, and myeloid and lymphoid cells. These cells affect the immune system independently or because of a complicated web of interconnections, which promotes disease development and immune evasion. Due to the importance of these factors in the onset of disease, various therapeutic strategies have been created that either target or improve the immunological processes that influence disease progression. The immune system has a role in the mechanism of action of multiple myeloma treatments. The main contributions of immune cells to the bone marrow microenvironment, as well as how they interact and how immune regulation might lead to therapeutic effects, are covered in this study.

## 1. Introduction

Multiple myeloma (MM) is described by clonally expanding plasma cells within the bone marrow (BM), monoclonal proteins detected in blood or urine, and end-organ damage [1,2]. MM is a cancer that is classified as a clonal B-cell malignancy and is currently incurable. The wide array of immunodeficiencies originating with this plasma cell dyscrasia is an intriguing aspect of the condition [3,4]. As per Cancer Stat Facts published by NIH, about 170,405 myeloma patients were reported from 2017 to 2019, amounting to 1.8% of all new cancer cases in the United States. The estimated new myeloma cases in 2023 are 35,730 (19,860 in men and 15,870 in women), while the number of fatal cases is 12,590 (7000 in males and 5590 in women) [5].

Plasma cells with malignant properties accumulate in bone marrow, causing multiple myeloma. The bone marrow is the soft matter inside bones where blood cells are made. Cancer cells obstruct the growth of healthy blood cells in the bone marrow. Cancer cells produce dysfunctional proteins rather than beneficial antibodies, leading to complications of multiple myeloma.

Bone marrow is thought to be a primary hematopoietic organ. The production and maturation of B lymphocytes take place in the bone marrow. The bone marrow contains the most long-lived plasma cells that can produce antigen-specific antibodies [6]. As a result, bone marrow supports humoral immune reactions. However, a growing body of research shows that immune cells like regulatory T cells, conventional T cells, B cells, dendritic cells, natural killer T (NKT) cells, neutrophils, myeloid-derived suppressor cells, and mesenchymal stem cells are actively functioning and moving around in the bone marrow. In addition, a few human cancers have a preset metastatic site in the bone marrow [7].

In recent years, new therapeutic options have been developed, and our understanding of the biology of the disease has improved [8,9,10]. Patients with myeloma increasingly receive immunomodulatory medications alone or in combination with routine treatment [11,12]. Additionally, adoptive T-cell treatments and immunotherapeutic approaches provide fascinating new research frontiers in myeloma treatment.

## 2. Role of Immune Cells in Multiple Myeloma (MM)

The immune system detects and destroys pathogens and foreign objects. An essential phase in developing myeloma is the evasion and inhibition of antitumor immunity. MM cells reproduce and proliferate almost exclusively in the BM niche, emphasizing how vital the microenvironment is for promoting cancer progression [13,14,15]. The BM microenvironment is highly vascularized and is made up of hematopoietic cells such as hematopoietic stem cells (HSCs), T and B lymphocytes, myeloid and natural killer (NK) cells, and dendritic cells [16]. The tumor microenvironment (TME) is a highly complex, constantly evolving phenomenon that facilitates bidirectional, mutually advantageous communication between malignant PCs and the BM milieu. TME acts as a protective niche, promoting tumor growth, increasing treatment resistance, and impairing immune surveillance. In MM, complex crosstalk between hematopoietic stem cells, myeloid cells, T and B lymphocytes, NK cells, erythrocytes, osteoclasts, (non-hematopoietic) osteoblasts, stromal cells, e.g., fibroblasts, endothelial cells, and acellular components, e.g., extracellular matrix and cytokines, growth factors, and chemokines produced by cellular components, plays a vital role in tumor progression and immune resistance [17,18]. Hypoxia alters TME and drives many forms of tumor invasion [19]. Hypoxic TMEs cause tumors to become more aggressive and metastatic [20]. Hypoxia-related parameters show predictive potential across malignancies [21]. The hypoxic environment changes the expression of genes that regulate metabolism and other functions. Furthermore, hypoxia signaling interacts with other biological pathways to alter cancer cell malignant behaviors and is intimately related to cancer cell proliferation, migration, invasion, and angiogenesis and affects cancer therapy [22].

### 2.1. T Cells

MM patients showed fewer CD4+ T cells in peripheral blood, connected to a reduced CD4/CD8 T-cell ratio compared to control healthy individuals [23,24]. In many studies, increased CD4/CD8 T-cell ratio was linked to CD8+ T cells with an activated cytotoxic phenotype (CD8+CD57+CD28−perforin+), but it was also linked to the growth of CD4+ T cells with an effector phenotype (CD28−CD4+). Favorable prognostic indicators have been linked to the surge in T-cell counts in MM patients. The immune system may be attempting to control the proliferation of plasma cells (PCs) utilizing these increased T-cell populations [25,26].

Perez-Andres et al. demonstrated a significantly higher rate of T-cell infiltration of the bone marrow in all patients with MM and plasma cell leukemia. Conversely, less CD4+CD8− and CD4−CD8− T cells were seen. Likewise, the cytotoxic/effector T-cell subsets of CD4+CD28− and CD8+CD28− were also diminished, and in most of the patient groups, TCR expression was elevated in both CD4+ and CD8+ BM T cells [27].

### 2.2. Helper T Cells

In adaptive immune responses, helper T cells are essential, and the unbalanced polarization of CD4 T-cell responses may significantly impact the progression of multiple myeloma. Though the specifics of the alterations vary, multiple investigations identify a dysregulated cytokine network in MM. Alpana Sharma et al. demonstrated changes in circulatory concentrations of cytokines produced from Th1 and Th2 cells during multiple myeloma [28]. In their study, they compared fifty healthy controls and sixty-two MM patients. They demonstrated that, in terms of serum concentrations of Th1 cytokines, IFN-γ was significantly decreased, and IL-2 was non-significantly higher, whereas IL-4 and IL-10 were considerably enhanced in terms of Th2 cytokines [28]. Th17 T helper cells are derived from naive CD4+ T cells and are implicated in the tumor immunological milieu [29,30]. Numerous investigations have demonstrated that, in comparison to healthy donors, MM patients have considerably higher levels of Th17 cells in PBMCs and serum concentrations of Th17-associated cytokines, such as IL-1b, IL-6, IL-17, IL-21, IL-22, and IL-23 [31,32,33]. Noonan et al. showed that the Th17 T-cell phenotype could be a critical predictor of lytic bone damage in multiple myeloma [34]. MM patients had higher levels of IL-17 than controls, possibly due to elevated levels of IL-6 in their bone marrow, which stimulates the generation of Th17 cells from CD4 naïve cells (as described in previous studies).

### 2.3. Regulatory T Cells (Tregs)

A vital subgroup of CD4 T-cells known as regulatory T-cells (Tregs) are typified by the CD4 + CD25 + Forkhead box P3+ (FoxP3+) phenotype and are capable of modulating peripheral tolerance as well as responses to foreign and tumor antigens. There is controversy over the role and quantity of Tregs in MM and their relationship to survival metrics, even though Tregs are raised in many forms of malignancy, including hematological malignancies. Treg-mediated inhibition of antitumor immunity has led to an increased frequency of Tregs, widely regarded as a sign of poor prognosis [35,36]. The peripheral blood of MM patients has been found to have a higher frequency of functional Tregs reported by multiple groups [37,38]. In peripheral blood from MM patients, Beyer et al. found that Tregs have a strong inhibitory effect and verified that the patients express higher levels of TGF-β and IL-10 than healthy participants or healthy donors [39].

Higher levels of Treg-associated markers, including CTLA-4, glucocorticoid-induced tumor necrosis factor receptor (GITR), and OX40, have been seen in the peripheral blood of MM patients, which may improve their suppressive function [39].

Tregs are separated into two subpopulations with distinct developmental origins: naïve Tregs (nTregs) and induced Tregs (iTregs). nTregs are progenitor cells in the bone marrow that commit to and mature in the thymus [40]. iTregs are negative selection cells created in the periphery from naïve T-cells. They are classified into type 1 regulatory T-cells (Tr1), which are induced by IL-10 [41,42] and T helper 3 (Th3) cells, which gain regulatory qualities via TGF-β [43,44].

Other lymphocyte subsets with regulatory qualities, such as gamma delta (γδ) T-cells, CD8 + T-cells, double negative cells, and regulatory-B-cells (Bregs), may not necessarily express CD4. Gamma delta T-cells are a small subset of T-cells distinguished by their γδ-T-cell receptors (TCR). They are primarily found in tissues and tumor sites where they exert suppressive activity towards naïve and effector T-cell responses. They also impede the proliferation and function of dendritic cells (DCs) [35]. Andrea Knight et al. found that γδ-T cells have potent cytotoxicity against primary multiple myeloma cells, making them a promising candidate for tumor immunotherapy [45].

### 2.4. B Cells

The proliferation of mature plasma cells in the bone marrow is a characteristic of MM, a B-cell malignancy. Circulating clonotypic CD19+ cells have been found in several investigations, indicating a development step before the PC. Although it varies greatly, circulating clonotypic CD19+ cells are usually modest. Though a small fraction of circulating clonotypic cells within the CD19 compartment is resistant to even high-dose chemotherapy, these cells react well to induction therapy [46].

B cells are traditionally recognized to positively influence immunological responses and inflammation through antibody production and enhance T-cell activation and proliferation via antigen presentation [47]. However, recent investigations in mice and humans have shown well-founded evidence of separate subsets of immunoregulatory B cells (Bregs/B10 cells) [48,49]. Bregs can maintain immune tolerance, suppress pathological autoimmune and inflammatory immune responses, and suppress responses during cancer immune surveillance by releasing anti-inflammatory mediators like in IL-10 [50,51]. Zhang et al. demonstrated that bone marrow-derived Breg cells inhibit NK cell-mediated ADCC against multiple myeloma cells in multiple myeloma patients by producing IL-10 [52].

B1 cells are a subset of B-cell lymphocytes participating in the humoral immune response [53]. B1a (natural antibody-producing cells) have been demonstrated to contribute to autoimmune disease by autoantibody generation [54,55], antigen presentation and activation of CD4 T cells [56], and cytokine production [57]. Some recent investigations have shown that B1a cells are considerably reduced in MM patients compared to healthy donors in both peripheral blood and bone marrow. The authors also assessed the impact of an immunomodulatory drug (lenalidomide) on B-cell subsets in MM. They analyzed B-cell subsets in PBMC from non-treated and lenalidomide-treated MM patients. They discovered that the lenalidomide-treated group showed significant improvement in B-cell subsets (increased B2 and decreased B1 cells) even before clinical response [58,59].

### 2.5. Macrophages

The myeloma microenvironment is rich in macrophages. Around 10% of the bone marrow (BM) of patients with multiple sclerosis (MM) is composed primarily of tumor-associated macrophages (TAMs) [60]. TAMs are derived from circulating monocytes that are drawn to the tumor site by cytokines such as VEGF, colony-stimulating factor (CSF)-1, chemokines, and CXC motif chemokine ligand (CXCL)-12 [61,62]. Common surface indicators for identifying macrophages are CD163 and CD206 [63]. Early research by Zheng et al. revealed that MM patients’ bone marrow had a higher concentration of CD68+ macrophages than healthy controls. By preventing caspase-3 and poly-ADP ribose polymerase (PARP) from being activated and cleaved and by preserving Bcl-xL levels in vitro, these macrophages promoted the growth and inhibited apoptosis of myeloma cells in the presence of treatment [60]. Furthermore, it was discovered that macrophages and mesenchymal stromal cells (MSCs) together promote the survival and proliferation of MM cells by producing IL-6 and IL-10 [64].

### 2.6. Myeloid-Derived Suppressor Cells (MDSCs)

MDSCs are a heterogeneous group of immature myeloid cells produced in the bone marrow [65]. In peripheral blood and bone marrow, a few publications described MDSCs in MM as CD33+ and CD11b+, low or HLA-DR low/-, and further subdivided into CD14 (monocytic MDSc) and CD15 (granulocytic MDSc) [65,66]. On the other hand, CD11b+ and Gr1+ expressions are typically used to identify murine MDSCs [67]. Generating reactive oxygen species (ROS) is necessary for MDSC-dependent immunosuppression. Several studies have reported that MDSCs derived from cancer patients and tumor-bearing mice generate enormous amounts of ROS [68,69], decreasing CD3ζ expression and antigen-specific T-cell proliferation [70]. Purified MDSCs from MM patients produced more Tregs than MDSCs from age-matched controls, according to research by Favaloro J et al. [66]. Various investigations reveal that MDSCs stimulate the clonal growth of antigen-specific natural T-regs and trigger the transformation of naïve T helper cells into inducible T-regs. This process relies on the interaction between CD40 and CD40L and the release of cytokines like TGF-beta, IL-10, and IFN-gamma [71,72]. The role of ROS molecules in the interaction of MDSCs and Tregs is unclear. The induction of Treg cells by macrophages involves ROS production; therefore, ROS deficiency might lead to reduced Treg induction and aggravate T-cell suppression [73]. Wang et al. demonstrated that bortezomib combined with dexamethasone resulted in a progressive decrease in the number of MDSCs. When MM cells and PBMCs were cocultured in the presence of bortezomib, M-MDSC levels were significantly reduced compared to MM cells and PBMCs alone [74]. Another study by Görgün et al. showed that exposure to bortezomib and lenalidomide in vitro did not affect the total quantity or immune suppressive capacity of MDSCs [75]. MDSCs, especially G-MDSCs, may interact with MM cells bidirectionally, implying that treatments targeting M-MDSCs may improve therapeutic outcomes in MM patients.

### 2.7. Natural Killer (NK) and Natural Killer T Cells (NKT Cells)

An integral part of the innate immune system, NK cells are cytotoxic lymphocytes linked to antitumor immunity [76]. Hematopoietic stem cells give rise to natural killer (NK) cells, which proliferate and acquire effector capabilities such as cytokine production and lysis capacity when exposed to IL-2 and IL-15 [77]. NKT cells constitute a conserved T-cell sub-lineage with unique properties, including reactivity for a synthetic glycolipid presented by CD1d and common natural killer cell markers such as NK1.1 in mice or CD161 in humans, expressing CD122 (IL-2Rb), Ly49 family receptors, and T-cell receptor [78,79]. The decrease in NK and NKT cells’ ability to secrete IFN-gamma is linked to the development of clinical myeloma, as revealed by Dhodapkar MV et al. [80,81]. Several studies showed that premalignant and early myeloma tumor cells had significantly elevated CD1d levels [82]. As the disease progressed, CD1d expression decreased and finally vanished completely, which was detrimental to myeloma cell survival [82].

### 2.8. Dendritic Cells (DCs)

DCs are significant antigen-presenting cells that can present autoantigens or foreign antigens [83,84]. DCs show expression on their surface, including DC-SIGN, CD1a, CD40, CD54, CD58, CD80, CD83, and CD86. Additionally, they have significant levels of MHC molecule class I and II expressions [85]. Typically, DCs are essential for plasma cell survival and differentiation [86,87]. A study by Brimnes et al. revealed that when MM patients were compared to healthy controls, there was a decrease in the quantity and expression of human leucocyte antigen (HLA) molecules on both myeloid and plasmacytoid DCs [88]. Moreover, MM patients’ CCR5, CCR7, and DEC205 expressions were lower than regular donors. According to Leone et al., DCs are said to accumulate in BM as MM progresses. The study demonstrated that DCs isolated from myeloma patients could engulf apoptotic myeloma cells, present them, and activate CD8+ T lymphocytes specific to tumors. Additionally, DCs inhibited the production of proteasome subunits in myeloma cells [89].

Myeloma pathology is also associated with plasmacytoid DCs (pDCs), the other major component of DCs. The production of normal plasma cells and antibody responses primarily depend on the secretion of IFN-gamma and IL-6 by pDCs [87,90]. MM patients have higher frequencies and quantities of BM pDCs, and studies by Chauhan et al. have demonstrated that pDCs give myeloma cells the ability to develop, survive, chemotaxis, and resist drugs [91]. pDC cells facilitate MM development and medication resistance. In vitro, MM-conditioned-pDCs lose their ability to release IFN-α, indicating a direct interaction between MM cells and pDCs, leading to a tumor-promoting phenotype [92]. Thus, to enhance myeloma development and avoid immune surveillance, MM cells regulate the function of pDCs via the CDH1 pathway. The molecular mechanisms driving pDC acquisition of these traits are unknown, contradicting findings on the amount of DCs in MM [89,91,93,94].

## 3. Regulation of Immune Cells by Immunotherapy in Multiple Myeloma

Immunotherapeutic agents used against multiple myeloma work in distinct ways. This section discusses some leading strategies and targeted actions that have emerged as promising treatments against MM.

### 3.1. Immunomodulatory Drugs

Immunomodulatory drugs (IMiDs) modulate the immune response by either stimulating the formation of serum antibodies (immunosuppressives) or inhibiting them (immunomodulators) [95]. IMiDs are a class of medications that exhibit many antimyeloma properties, including immunomodulation, anti-angiogenic, anti-inflammatory, and antiproliferative effects [96,97].

IMiDs mediate antimyeloma action via direct and indirect mechanisms [98]. The proliferation and survival of myeloma cells were not significantly affected by first-generation IMiDs, such as thalidomide. These medications were employed initially as hypotonic sedatives and were later withdrawn because of teratogenic consequences [99]. However, because of their anti-angiogenic and immunomodulatory qualities, they were reinstated as a therapy option for multiple myeloma. Singhal et al. treated eighty-four patients with refractory myeloma, in which seventy-six patients relapsed after a high dose of chemotherapy received oral thalidomide as a single agent for a median of 80 days. Authors observed durable responses in some patients with multiple myeloma, including those who relapse after high-dose chemotherapy [100]. Four distinct phase III trials compared the use of thalidomide in conjunction with melphalan or prednisone in transplant-eligible patients. One of the experiments included thalidomide maintenance until relapse [101]. In MM cells, second-generation IMiDs can directly cause cell cycle arrest and apoptosis [102,103]. IMiDs are the cornerstone of myeloma treatment, and in clinical trials, they typically serve as the gold standard to which other medications are added. In phase II research, Niesvizky et al. demonstrated that adding clarithromycin to lenalidomide and dexamethasone (BiRD) resulted in a greater overall response rate [104]. In another trial, lenalidomide demonstrated potential activity as a maintenance medication after autologous stem cell transplantation. McCarthy et al. found a median duration to advancement of 46 months versus 27 months [105]. Monoclonal antibodies, steroids, protease inhibitors, and epigenetic regulators are combined with IMiDs [106,107]. Primary medications used to treat MM are listed in Table 1.

IMiDs can interact with various cells, including immune cells, osteoclasts, and bone marrow stromal cells, which interfere with the interaction between the MM cells and the bone marrow milieu, explaining the indirect mechanisms. The interaction between MM cells and the bone marrow microenvironment promotes cell growth, survival, migration, and drug resistance. IMiDs work against MM clone proliferation by acting as anti-angiogenic and anti-inflammatory agents. They also mediate cytokine production and surface adhesion molecule expression on MM and bone marrow stromal cells [130,131]. According to various reports, IMiDs suppress the expression of bFGF and VEGF, prevent the synthesis of TNF-α, IL-1, IL-6, and IL-12, delay the development of osteoclasts, and alter the activity of some immune system cells [132,133].

**Figure 1:**
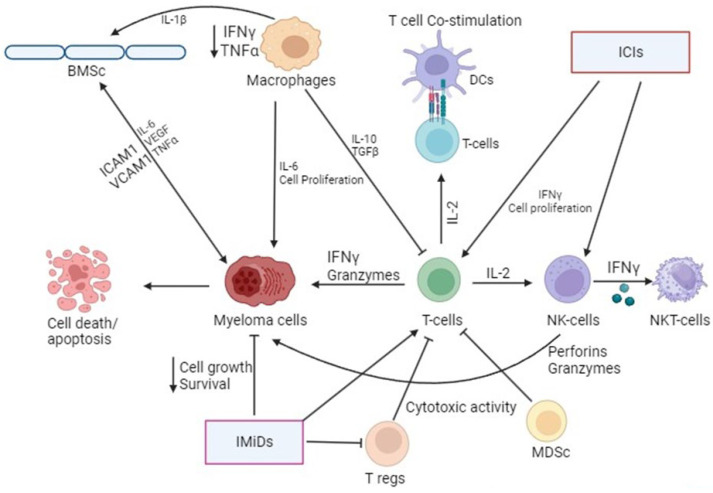
Graphical representation of different immune cells and their regulation in myeloma. Modulation of immune cell function by immunotherapy agents (IMiDs—immunomodulatory drugs; ICIs—immune checkpoint inhibitors) is used for multiple myeloma treatment.

IMiDs differ from other antimyeloma medications solely by their immunomodulatory effect. Thalidomide is less effective than second-generation IMiDs in promoting CD4+ and CD8+ T-cell co-stimulation (Figure 1). Tregs are also crucial for maintaining the body’s immunological tolerance and share many molecular signaling pathways with conventional T cells, especially cytotoxic T cells, which are principal mediators of tumor immunity [132,134,135]. The potential synergistic effect of this immunomodulatory function with emerging immunotherapeutic drugs like checkpoint inhibitors and monoclonal antibodies makes it intriguing.

NK and NKT cells are enhanced by IMiDs [136]. They promote NK cell proliferation in MM patients responding to therapy and enhance NK cell-mediated cytotoxicity and antibody-dependent cellular cytotoxicity (ADCC) induced by triggering IL-2 production from T cells [137].

### 3.2. Immune Checkpoint Inhibitors (ICIs)

The survival rates of numerous solid tumors have significantly increased with immune checkpoint inhibitors [138]. As a hematologic malignancy, MM has a clear indication that the tumor’s pathophysiology was influenced by a compromised immune system [139]. ICIs cause cancer cells to undergo apoptosis, stop their proliferation, and block receptors that inactivate lymphocytes [140]. Programmed cell death protein 1 (PD-1), cytotoxic T-lymphocyte-associated protein 4 (CTLA-4), and programmed death-ligand 1 (PD-L1) are different types of ICIs. T cells, a subset of white blood cells directly supporting the body’s immune system in battling illness, have PD-1 on their surface. Blocking PD-1 improves the immune system’s ability to eradicate the disease by preventing the body from eliminating cancer cells [141,142]. PD-L1 on MM cells inhibits the activation potential and triggers T-cell activation [142]. CTLA-4 is a receptor present on the surface of T cells. CTLA-4 expression was seen upon activation of T cells, including memory and regulatory T-cells [143]. It has been demonstrated that patients with MM have higher levels of CTLA-4 and PD-L1 expression in their bone marrow microenvironment [144]. Additional checkpoint inhibitors that target T-cell immunoglobulin 3 (TIM3), T-cell immunoreceptors with immunoglobulin and ITIM domains (TIGIT), and lymphocyte activation gene-3 (LAG3) are under investigation [145].

A phase I multicenter, multi-cohort study in patients with RRMM that combined the PD-1 inhibitor pembrolizumab, lenalidomide, and low-dose dexamethasone reported that 62 patients had a 44% ORR. Another study confirmed the efficacy of bonadomide and lenalidomide in enhancing ICIs in myeloma cells. In contrast, a combination of dexamethasone, lenalidomide, and the PD-L1 antibody pembrolizumab was effective in patients with lenalidomide-refractory myeloma, with an objective response rate of 76% [146].

### 3.3. Chimeric Antigen Receptor (CAR)-T Cell Therapy

Despite a broad range of approved treatments, relapsed/refractory multiple myeloma (RRMM) continues to pose a serious clinical challenge. T-cells engineered with chimeric antigen receptors (CARs) have revolutionized the treatment of patients with hematologic malignancies. CAR-T cells targeting CD-19 B-cell lymphoma cells have demonstrated significant efficacy in treating acute lymphoblastic leukemia and lymphoma [147]. The treatment of MM with CAR T cell therapy has shown considerable promise [148]. The B-cell maturation antigen is the most often tested target for MM. Host cells modified to express an antigen recognition domain capable of binding a specific target for tumors are called CAR-T cells [149,150]. CAR-T receptors are manufactured transmembrane proteins containing antigen recognition domains linked by a linker, followed by costimulatory and T-cell activation domains [151].

#### 3.3.1. B-Cell Maturation Antigen (BCMA) CAR-T

The highly selective expression of B-cell maturation antigen (BCMA) in malignant plasma cells makes it a novel target for MM treatment. BCMA is also known as tumor necrosis factor receptor superfamily member 17 (TNFRSF17) or CD269. A crucial element of plasma cell homeostasis, this transmembrane protein controls B-cell development and differentiation into plasma cells [152]. BCMA’s correlation with plasma cell survival satisfies the functional dependency condition for an ideal target [153]. The higher levels of its ligands facilitate the proliferative and anti-apoptotic behavior of MM, B-cell activating factor (BAFF), and a proliferation-inducing ligand (APRIL) [154]. In the first-in-human BCMA CAR-T study, researchers at the National Cancer Institute (NCI) employed CD28 for the costimulatory domain, retrovirus vector for transduction, and murine scFv for antigen recognition [155]. Their remarkable 81% objective response rate (ORR) was reported. Subsequently, the outcomes of other BCMA CAR-T clinical trials were released. Selected clinical studies of BCMA CAR-T are summarized in Table 2.

#### 3.3.2. Non-BCMA CAR-T

In patients with RRMM, CAR-T cell treatments with BCMA have produced extremely encouraging outcomes. Studies conducted on patients receiving BCMA CAR-T treatments over an extended period showed no relapses [166]. CAR-T cell treatments targeting non-BCMA antigens have demonstrated efficacious outcomes in MM patients [167]. When combined with BCMA, new targets exhibit extremely encouraging results. Non-BCMA targets include CD138 (syndecan-1), CD38, NY-ESO-1, NKG2D ligands, SLAMF7/CS1, CD56, Lewis Y, and GPRC5D.

Cluster of Differentiation 19 (CD19) is a transmembrane protein encoded by the CD19 gene. All human B-cell lineages express CD19. The myeloma plasma cell surface exhibits a low expression of CD19; CD19+ cells may be a subgroup of stem cells in patients with myeloma. Several studies demonstrated that CD19 CART cells with anti-BCMA therapies showed promising efficacy in treating MM [168]. Recently, G-Protein Coupled Receptor 5D (GPRC5D) has been found as another possible target for MM because this antigen is expressed at high levels on malignant MM cells regardless of BCMA distribution and only at low levels on B cells, healthy plasma cells, and hair follicles [169]. MM cells exhibit high expression of CD138, commonly known as syndecan-1. According to a study, CD138-directed CART cells can eradicate primary myeloma cells in vivo and in vitro [170]. The New York Esophageal Squamous Cell Carcinoma 1 (NY-ESO-1) testis gene is highly immunogenic and often expressed in many cancer types [171]. TCR-engineered T cells specific to NY-ESO-1 have produced positive therapeutic outcomes in MM patients [172].

The signaling lymphocytic activation molecule (SLAM) receptor family includes the SLAMF7 (CD319, CS-1) receptor. SLAMF7 can influence various immune cell-specific functions in several cell types [173]. The first FDA-approved antibody for the treatment of MM was elotuzumab, a humanized monoclonal antibody targeted to SLAMF7. Emotruzaba, in conjunction with lenalidomide or pomalidomide, has demonstrated therapeutic effectiveness in patients with relapsed multiple myeloma [174].

NKG2D (natural-killer group 2, member D) receptor plays a vital role in protecting the host from infections and cancer. NKG2D is one of the best-characterized activating immune receptors associated with tumor immunosurveillance [175,176]. Both primary and metastatic cancer cells frequently upregulate stress-induced ligands closely related to MHC class I [177]. NKG2D CAR T-cell therapy showed a therapeutic effect on MM, lymphoma, and ovarian cancers in vivo [178]. Table 3 summarizes the selected non-BCMA-targeted CAR-T clinical studies.

#### 3.3.3. CAR-T Therapy Limitations and Toxicities

Despite its current success rate, CAR-T treatment is associated with substantial toxicities. The most prevalent adverse effects associated with CAR-T treatment, such as cytokine release syndrome (CRS) and immune effector cell-associated neurotoxicity syndrome (ICANS), continue to be hurdles to its usage. CRS is the most common type of toxicity associated with CAR-T treatment. Symptoms typically appear within the first week following CAR-T cell injection and last 7 to 8 days [188]. CRS is considered a reversible consequence of CAR-T treatment, occurring in 42% to 100% of patients. It manifests with varied intensity, with 0 to 46% of patients presenting severe CRS symptoms and 0 to 9.1% of cases proceeding to death [189]. ICANS, like CRS, is a transient issue that occurs in the majority of patients following CAR-T infusion. It is less common and more typically delayed than CRS, with an incidence ranging from 3 to 64% and 0 to 54% of cases presenting indications of severe disease [190]. It is a clinical and neuropsychiatric illness manifestation that develops between days to 2–3 weeks of CAR-T treatment administration [191]. Toxicology prevention and management strategies have been presented from a variety of perspectives. Optimization of dosing schemes is a realistic clinical strategy, while laboratory strategies involve creating less toxic CAR structures, which could considerably increase the safety of CAR T-cell treatment. However, further research is necessary before these treatments can be extensively used in clinical settings.

## 4. Limitations of Immunotherapy

Developing inherent or acquired resistance is a significant hurdle in cancer immunotherapy research. Cancer cells have a variety of methods to avoid immunosurveillance, which causes resistance to immunotherapy, such as checkpoint inhibitors, CAR-T cell treatment, and recombinant cytokines [192]. Immunotherapies are commonly limited by Immune-Related Adverse Events (irAEs), which include impervious actuation and an incendiary reaction against the host’s healthy tissues. Although irAEs aim to forecast, evaluate, and cure, the ideal outcome is resistant actuation against host development [193,194].

Despite solid response rates in numerous BCMA-targeted CAR T-cell treatments, response durability remains a clinical hurdle in the treatment of MM. A significant number of patients develop recurrence [195]. Other challenges include the inability to predict the efficacy of the therapy and the patient’s response, the need for additional biomarkers, the improvement in immunity in opposition to malignant immunotherapies, the lack of up-to-date medical assessment plans to determine sufficiency, and high therapy costs.

## 5. Future Perspectives in the Field of Immunotherapy

Checkpoint inhibitors, antibody-based medicines, adoptive cell therapy, BCMA, and non-BCMA CAR-T treatments are all promising advances in treating multiple myeloma [196,197]. Ongoing clinical trials are testing bispecific T cell antibodies in conjunction with other therapies, such as IMiDs and PIs, with the potential for further anti-myeloma action. Furthermore, current research investigating the combination of BCMA targeting diverse antigens on plasma cells is particularly intriguing, hinting at the prospect of pure immunotherapy for MM, eliminating the toxicity of traditional therapy. To reduce the risk of recurrence in multiple myeloma after CAR T-cell therapy, researchers are concentrating on new antigens that can be targeted alone or in combination with BCMA [198]. Furthermore, pharmacological medications can increase the density of target antigens on MM cells, thereby enhancing patient outcomes. Exploring the impact of specific genomic and transcriptomic markers, such as T-cells and RNA editing signatures, on surface target expression may reveal new paths for immune targeting in MM patients. Ongoing research aims to discover techniques to increase target antigen expression, improve immunotherapy durability, and overcome immunosuppressive obstacles in the tumor microenvironment. These improvements show promise for the future of multiple myeloma therapy.

## 6. Summary

This study investigated the intricate relationship between immune cells and cancer and their function in halting the formation of tumors and the disease. Understanding the potential of immune-based therapy and cancer therapies is facilitated by this review. Identification of antigens associated with myeloma and strategies to reverse tumor-mediated immune suppression are critical components of effective immunotherapy. Regression of the disease seems to be highly induced by antibody therapy, either by itself or in combination with other anti-MM drugs. While checkpoint blocking has been shown to have potential as a cancer treatment, combined therapy may be necessary for checkpoint blocking to be effective in multiple myeloma. The immune system has developed into a potent tool because of its capacity to recognize cancer cells, capture tumor heterogeneity, and supply memory to prevent a recurrence. For MM patients, the long-term prognosis is expected to be significantly improved by developing cell therapies in combination with immunomodulatory drugs, immune checkpoint inhibitors, and CARTs.

## Figures and Tables

**Table 1:** Drugs used in the treatment of multiple myeloma.

Drugs	Mode of Action	Route of Administration	Overall Response Rate (ORR)	References
	**Immunomodulatory Drugs**			
Thalidomide	Binds to CRBN and triggers proteasomal degradation of IKZF1 and IKZF3, leading to direct anti-multiple myeloma activity and immunomodulatory and stromal cell effects.	Oral	28%	[96,108]
Lenalidomide	Bind CRBN, the substrate adaptor for the CRBN-CRL4 E3 ubiquitin ligase, and modulate the enzyme’s substrate specificity. These drugs induce the degradation of two lymphoid transcription factors, IKZF1 and IKZF3, leading to dramatic clinical efficacy in multiple myeloma.	Oral	25–27%	[109]
Pomalidomide	Degrades the IKZF1 and IKZF3 proteins more potentially.	Oral	30–35%	[110]
Mezigdomide	The drug is designed to achieve rapid, potent, and deep degradation of Ikaros and Aiolos, key transcription factors in hematopoietic cell development and differentiation.	Oral	40%	[111]
	**Proteasome inhibitors**			
Bortezomib	Primarily acts through plasma cell depletion, reducing antibody production to T-dependent antigens.	Intravenous (IV) or subcutaneous injection	38%	[112,113]
Carfilzomib	The drug irreversibly binds with the proteasome and inhibits its chymotrypsin-like activity.	Intravenous infusion	23%	[114]
Ixazomib	Reversibly binds and inhibits the chymotrypsin-like proteolytic (β5) site of the 20S proteasome, reducing the tumor progression.	Oral	78%	[115]
	**Corticosteroids/Steroids**			
Dexamethasone	Agonist to glucocorticoid receptor. It prevents white blood cells from migrating to locations where malignant myeloma cells are causing damage and reduces swelling and inflammation.	Oral or intravenous	90%	[116]
Prednisone	Agonist to glucocorticoid receptor.	Oral	10%	[117,118]
	**Alkylating drugs**			
Melphalan	Melphalan induces inter- or infrastructural DNA crosslinks and DNA–protein crosslinks. Increases tumor-specific CD4 + T-cell responses and inhibits BM stromal cell expression of IL-6.	Intravenous (IV) and oral	82%	[119,120]
Cyclophosphamide	The formation of DNA crosslinking alters cytokine expression in the BM microenvironment, causing MM cell death/apoptosis.	Intravenous (IV) and oral	58.1%	[121]
Bendamustine	Formation of DNA crosslinking. Checkpoint controls inhibition, causing a mitotic catastrophe.	Intravenous (IV)	55%	[122,123]
	**Monoclonal Antibodies**			
Elotuzumab	Drug targets the signaling lymphocytic activation molecule F7 (SLAMF7). It directly activates natural killer cells, enhancing their ability to kill myeloma cells.	Intravenous infusion	79%	[124,125]
Daratumumab	It works by targeting the protein CD38 on myeloma cells. It helps to slow or stop the progression of multiple myeloma.	Intravenous infusion or subcutaneous injection	81%	[126,127]
Isatuximab	Anti-CD38 monoclonal antibody exerts antimyeloma activity through several mechanisms of action, such as antibody-dependent cell-mediated cytotoxicity, complement-dependent cytotoxicity, and direct induction of apoptotic cell death.	Intravenous infusion	23.8%	[107]
	**Epigenetic inhibitors**			
Panobinostat	It is an HDAC inhibitor used in the treatment of relapsed and refractory multiple myeloma.	Oral	24.8%	[128,129]

**Table 2:** Selected BCMA CAR-T clinical trials.

Trial Number	Name	Phase	Number of Patients	Overall Response Rate (ORR)	Sponsors	References
NCT03548207	CARTITUDE-1	2	113	89%	Janssen Research & Development, LLC	[156]
NCT03338972	FCARH143	1	12	100%	Fred Hutchinson Cancer Center	[148]
NCT05393804	bb2121	2	Recruiting	NA	Memorial Sloan Kettering Cancer Center	[157]
NCT02658929	bb2121 or Ide-cel	1	33	85%	Bluebird Bio and Celgene	[158]
NCT02215967	CAR-BCMA	1	24	81%	National Cancer Institute (NCI)	[155]
NCT03361748	KarMMa	2	140	73%	Celgene	[158,159]
NCT04181827	CARTITUDE-4	3	419	76%	Janssen Research & Development, LLC	[160,161]
NCT03090659	LEGEND-2	1	57	88%	Nanjing Legend Biotech Co.	[162]
NCT03430011	Orva-cel	1/2	44	82%	Juno Therapeutics, Celgene	[163,164]
NCT04155749	NA	1	13	60–75%	Arcellx, Inc.	[165]

**Table 3:** Selected non-BCMA-targeted CAR-T clinical trials.

Trial Number	Name	Phase	Number of Patients	Sponsors	References
NCT02135406	CTL019	1	12	University of Pennsylvania	[179]
NCT03767725	NA	1	Recruiting	Shenzhen Second People’s Hospital	[73]
NCT03706547	NA	1	Unknown	Peng Liu	[180]
NCT02203825	NA	1	6	Celyad Oncology SA	[181]
NCT03018405	THINK	1	16	Celyad Oncology SA	[182]
NCT04674813	CC-95266	1	21	Juno Therapeutics, a Subsidiary of Celgene	[183]
NCT03710421	NA	1	Recruiting	City of Hope Medical Center	[184]
NCT03778346	NA	1	Recruiting	The Sixth Affiliated Hospital of Wenzhou Medical University	[185]
NCT01716364	NA	1	6	Peter MacCallum Cancer Centre, Australia	[186]
NCT03464916	CAR2 Anti-CD38 A2	1	Unknown	Sorrento Therapeutics, Inc.	[187]

## Data Availability

Not applicable.
